# Exploring the Effects of Vitamin D and Vitamin A Levels on the Response to COVID-19 Vaccine

**DOI:** 10.3390/vaccines11091509

**Published:** 2023-09-21

**Authors:** Hassan M. Kofahi, Baha’ R. Badran, Refat M. Nimer, Ali M. Atoom, Shefa’ M. Al Hersh

**Affiliations:** 1 Department of Medical Laboratory Sciences, Faculty of Applied Medical Sciences, Jordan University of Science and Technology, P.O. Box 3030, Irbid 22110, Jordan; brbadran20@ams.just.edu.jo (B.R.B.); rmnimer@just.edu.jo (R.M.N.); smalhersh16@ams.just.edu.jo (S.M.A.H.); 2 Department of Medical Laboratory Sciences, Al-Ahliyya Amman University, Amman 19328, Jordan; a.atoom@ammanu.edu.jo

**Keywords:** vaccine, COVID-19, immune response, vitamin D, vitamin A

## Abstract

COVID-19 vaccines were developed at an unprecedented speed in history. The factors affecting the response to COVID-19 vaccines are not clear. Herein, the effects of vitamin D and vitamin A (retinol) levels on the response to the BNT162b2 vaccine were explored. A total of 124 vaccine recipients were recruited from the general population attending vaccination centers in Irbid, Jordan. Blood samples were collected immediately before receiving the first vaccine dose (D0) and three weeks later (D21). Baseline (D0) levels of 25-hydroxyvitamin D [25(OH)D], retinol, and SARS-CoV-2 S1 IgG antibodies were measured with ELISA. The response to the BNT162b2 vaccine was tested by measuring the levels and avidity of SARS-CoV-2 S1 IgG antibodies on D21. The participants were divided into two groups, unexposed and exposed, based on the D0 SARS-CoV-2 antibody results. No significant correlation was found between the levels of 25(OH)D or retinol and the levels, avidity, or fold increase of antibodies in both groups. Similarly, no significant difference in antibody response was found between 25(OH)D status groups, retinol status groups, or combined status groups. These findings show that the baseline vitamin D or vitamin A levels have no effect on the short-term response to a single dose of BNT162b2 vaccine.

## 1. Introduction

The coronavirus family member, severe acute respiratory syndrome coronavirus 2 (SARS-CoV-2), caused more than 760 million confirmed cases of coronavirus disease 2019 (COVID-19) and more than 6.9 million deaths as of Aug 2023 [1]. Since the early days of the pandemic, it was believed that developing an effective vaccine is important for reducing the numbers of infections and mortalities. Hence, many COVID-19 vaccines were developed, tested, and authorized in an unprecedented speed in human history. Today, several COVID-19 vaccines are authorized for use and more than 13 billion vaccine doses were administered globally [1].

BNT162b2 (Pfizer-BioNTech, Comirnaty) was the first COVID-19 vaccine to receive emergency use authorization in the United States and Jordan [2]. In addition, this vaccine was the first mRNA vaccine to be administered on a wide scale in history. BNT162b2 contains an mRNA encoding a modified version of the full-length spike protein of SARS-CoV-2 that has stabilized it in its prefusion conformation [3]. After injection, the mRNA is taken by the cells and translated into S protein, which stimulates the immune system to generate neutralizing antibodies and memory cells [4].

Vitamin D is known to regulate calcium and phosphorus metabolism, thus playing a key role in bone formation [5]. The expression of vitamin D receptors (VDRs) and vitamin D activating enzyme is not limited to the cells involved in mineral and bone homeostasis [6]. Therefore, vitamin D is suggested to play multiple roles in the body including the regulation of the immune responses [6,7]. Besides improving skeletal health, having sufficient vitamin D levels is associated with reduced risk of multiple diseases including cancer, cardiovascular diseases, type II diabetes mellitus, and autoimmune diseases [8]. Vitamin D was also shown to have antibacterial functions by stimulating several cell types to produce antibacterial factors and to induce autophagy [9].

Due to its immunomodulatory functions, the effect of vitamin D on antibody responses to certain vaccines was investigated previously. For example, the recipients of vitamin D supplementation generated higher titers of tetanus toxoid-specific IgG antibodies in response to vaccination compared with the placebo control group [10]. Also, a higher frequency of non-respondents to hepatitis B vaccine was found among the vitamin D deficient group (25-hydroxyvitamin D [25(OH)D] < 10 ng/mL) compared to the vaccine-recipients with normal 25(OH)D levels [11]. In contrast, no association was found between serum vitamin D and the immunogenicity of influenza vaccines in elderly and HIV-infected populations [12,13,14]. However, a strain-specific positive effect of vitamin D on the response to the influenza vaccine was reported in a systematic review [15]. This review found lower seroprotection rates to A/H3N2 and B strains of influenza in vitamin D deficient vaccine recipients compared to the recipients with normal vitamin D levels.

The effect of vitamin D on the responses to COVID-19 vaccines is still not clear as conflicting results were reported by different groups. For example, a randomized controlled trial found that the supplementation of adults having sub-optimal levels of vitamin D had no effect on the risk of breakthrough SARS-CoV-2 infections, the titers of antibodies, or IFN-γ levels after receiving two doses of either ChAdOx1 nCoV-19 (Oxford−AstraZeneca) or BNT162b2 vaccines [16]. Meyers et al. found no significant differences in SARS-CoV-2 antibody levels or 50% pseudoneutralization titers between the vitamin D status groups of nursing home residents and staff who were vaccinated with two doses of BNT162b2 [17]. Similarly, Chillon et al. reported that vitamin D status had no significant effect on SARS-CoV-2 IgG levels and neutralization potency in response to two doses of the BNT162b2 vaccine [18]. Furthermore, Zelini et al. found no significant effect of baseline vitamin D level on the short-term (21 days) response to the BNT162b2 vaccine; however, this group found a significant correlation between the baseline vitamin D levels and the long-term response (six months after the second dose) [19]. In agreement with this, di Filippo et al. found significantly lower levels of SARS-CoV-2 antibodies 9 months after receiving the second BNT162b2 vaccine dose in vitamin D deficient individuals [20]. In contrast, Piec et al. investigated the effects of vitamin D status on the response to one dose of the BNT162b2 vaccine in a cohort of healthcare workers and showed a significant positive effect of the vitamin D status on the peak levels of SARS-CoV-2 antibodies, which was achieved at 3.2 weeks after vaccination on average [21].

The vitamin A group includes retinol, retinyl ester, and provitamin A [22]. Vitamin A compounds promote the growth and development and help in protecting against cancer [22,23]. Furthermore, 11-cis-retinal, an active vitamin A derivative, associates with a protein to form a complex known as rhodopsin. This complex plays a crucial role in light sensing by the retina. Thus, a vitamin A deficiency was found to be a main cause of night blindness [24]. Vitamin A is also necessary for normal reproductive functions in both males and females [25]. The effects of vitamin A on the immune responses were studied since early in the 20th century. Vitamin A deficiency was reported to associate with impaired functions of the innate and adaptive immunity and with increased susceptibility to infections [26,27]. Hematopoiesis was found to be influenced by the levels of vitamin A and its derivatives, as it is required for the normal differentiation and development of granulocytes, monocytes, lymphocytes, and erythrocytes [23]. In addition, the functions of macrophages, neutrophils, and natural killer cells, as well as the integrity of the epithelial barriers, were found to be impaired in vitamin A deficient individuals [28]. Similarly, vitamin A improves some adaptive immunity components including T cell counts, T cell functions, and the antibody response to some antigens [29]. Vitamin A supplementation was shown to improve the outcomes of several viral, bacterial, and protozoan infections including human papillomavirus, measles, diarrheal diseases, respiratory infections, human immunodeficiency virus, and malaria [29,30,31].

Vitamin A levels were reported to influence the responsiveness to vaccines. For example, the immunogenicity of the pneumococcal vaccine (Prevnar-13) improved significantly with vitamin A supplementation in a mouse model [32]. Vitamin A supplementation was reported to have a positive effect on the seroconversion rates and on measles antibody titers after vaccination [33,34,35]. Moreover, positive correlations between vitamin A levels and the immunogenicity of other vaccines were reported including the oral polio vaccine, tetanus vaccine, and hepatitis B vaccine [36,37,38]. To the best of our knowledge, the effect of vitamin A on the response to COVID-19 vaccines was not reported before.

Phase III clinical trial results showed that the efficacy of BNT162b2 vaccine in preventing COVID-19 is 95% [39]. However, variations in the response to the vaccine between individuals were noted. The causes of these variations are not yet fully understood [40]. Many groups investigated the effects of different demographic and clinical host factors on the responsiveness to the vaccine. Vitamins were shown to affect the immunogenicity of other vaccines and may influence COVID-19 vaccine immunogenicity as well. This study aims to investigate the effects of vitamin D and vitamin A on the response to the BNT162b2 vaccine by exploring the correlations between SARS-CoV-2 S1 antibody levels or avidity and the baseline levels of the two vitamins. Moreover, we explored the effects of several demographic and clinical factors.

## 2. Materials and Methods

### 2.1. Study Design

The study was approved by the Institutional Review Boards (IRB) of Jordan University of Science and Technology (Ref.: 42/140/2021, date: 27 May 2021) and the Jordanian Ministry of Health (Ref: Moh/REc/2021/146, date: 16 August 2021). Study participants were recruited from the general population attending two vaccination facilities at Jordan University of Science and Technology and Al-Hassan Sports City in Irbid, Jordan, from June to October 2021. During this period, the Jordanian health authorities allowed COVID-19 vaccines only for recipients aged 30 years and older. Each participant signed an informed consent prior to their participation. A questionnaire was then filled out by each participant with the help of trained research assistants, and a blood sample was withdrawn. All the study participants received BNT162b2 (Pfizer-BioNTech) COVID-19 vaccine. Immunocompromised individuals and pregnant women were excluded from the study.

Blood samples were collected at two time points. The first blood sample was collected from each participant on day 0 (D0) immediately before receiving the first vaccine dose. The second blood sample was collected on day 21 (D21), three weeks after receiving the first vaccine dose and before receiving the second vaccine dose. Serum was separated from each sample, aliquoted into at least 4 microcentrifuge tubes, and stored at −80 °C until the day of analysis.

The questionnaire collected demographic and clinical information from each participant including age, sex, weight, height, diseases history, smoking history, and usage of vitamin A and vitamin D supplements. Body mass index (BMI) was calculated for each participant according to the following formula: BMI = Weight (Kg)/height^2^(meter). Participants were classified into BMI categories according to the WHO recommendations [41]. Participants with BMI values < 25 Kg/m^2^ were considered normal, BMI values 25–30 Kg/m^2^ were considered overweight, and BMI values > 30 Kg/m^2^ were considered obese.

### 2.2. Vitamins and Antibody Measurement and Classification

The levels of 25(OH)D and retinol were measured in D0 serum samples with competitive ELISA using commercially available kits (Cat. Numbers: MBS580159 and MBS2000356, respectively, MyBioSource, San Diego, CA, USA). The tests were performed according to the manufacturer’s instructions.

Serum SARS-CoV-2 S1 IgG antibody levels were quantified at the two time points (D0 and D21) using a commercially available indirect ELISA kit (MBS398013, MyBioSource, San Diego, CA, USA) and according to the manufacturer’s instructions. Antibody levels of less than 5 IU/mL were considered negative according to the manufacturer’s recommendations.

The status of vitamin D was classified using the following: 25(OH)D levels of <20 ng/mL were considered deficient, levels between 20 and 29.9 ng/mL were defined as insufficient, and levels of 30 ng/mL and more were considered normal [42]. On the other hand, retinol levels less than 200 ng/mL were considered deficient [43].

### 2.3. Antibody Avidity Assay

The commercially available SARS-CoV-2 S1 IgG ELISA kit (MBS398013, MyBioSource San Diego, CA, USA) was used in conducting this test. The avidity of the D21 SARS-CoV-2 S1 antibodies was determined as described previously by Pichler et al., with minor modifications [44]. Briefly, each sample was run in duplicate in two parallel SARS-CoV-2 S1 ELISA microplates. One microplate was run according to the manufacturer’s instructions. The other microplate was run according to a modified protocol in which urea was added to the washing buffer of the last step of the first round of washing at a concentration of 5.5 M. In this step, parallel wells were filled with 300 μL of either the unmodified washing buffer or the urea-containing washing buffer and incubated for 10 min at room temperature. All the other steps of the protocol were performed according to the manufacturer’s instruction. After completion, the optical density (OD) for urea-treated and untreated wells was measured and the avidity index for each sample was calculated with the following equation:

$$Avidity\;Index\;\left(\%\right)=\frac{OD\;(ureatreatedwell)}{OD\;(untreatedwell)}\times 100\%$$

### 2.4. Statistical Analysis

The data were analyzed using GraphPad Prism 6 software (GraphPad Software, Inc., USA) and the Statistical Package for the Social Sciences version 26 (SPSS) software (IBM Inc., Armonk, NY, USA). Numerical variables were presented as means ± standard deviations (SD) while categorical variables were presented as counts and percentages. The numerical data are presented in this article according to the recommendations of Cole [45]. Unpaired *t*-test and one-way ANOVA were used to compare means between groups. Two-tailed *p*-values < 0.05 were considered statistically significant. Pearson’s correlation was used to analyze the correlation between D21 antibody levels or avidity and D0 vitamins’ levels. The outliers were identified as having values of more/less than mean ± 3SD and were excluded from the correlation analyses and from the comparisons of means between groups.

## 3. Results

### 3.1. Characteristics of the Study Sample

A total of 311 participants were recruited into the study and donated the first (D0) blood sample. However, only 124 participants returned for the second (D21) blood sample and were included in the analysis. The mean age for the study population was 43.8 ± 9.2. Females constituted 47.6% of the study sample. The mean BMI for the study sample was 27.7 ± 6.3 which falls in the overweight category according to the WHO classification system. A total of 26 (21%) participants reported having at least one chronic disease at the time of vaccination. Hypertension was the most frequently reported chronic disease in the study sample and was reported by 20 (16%) participants; 14 participants (11%) reported having diabetes, and 6 participants (4.8%) reported having cardiac diseases. The detailed characteristics of the study sample are summarized in Table 1.

SARS-CoV-2 antibody levels were measured on two time points for each participant: D0 and D21. The presence of detectable levels of SARS-CoV-2 antibodies (>5 IU/mL) on D0 was used as an indication of previous exposure to the virus. The results showed that 85 participants (69%) were not previously exposed to the virus (Table 1).

The serum level of 25(OH)D was tested on D0 before vaccination, and the mean ± SD level for the study sample was 14.3 ± 7.4 ng/mL. The serum levels of vitamin D were deficient (<20 ng/mL) in 85%, insufficient (20–30 ng/mL) in 12%, and normal (>30 ng/mL) in only 3.1% of the study sample (Table 1). No significant differences in 25(OH)D levels were found between the participants with or without a history of chronic diseases (*p* = 0.98) or between the BMI groups (*p* = 0.09). In contrast, the mean level of 25(OH)D in male participants was significantly higher than in females (15.9 ± 5.2 vs. 11.1 ± 5.6, *p* < 0.001) (Supplementary Figure S1).

Retinol serum level was also tested on D0. The results showed that the mean ± SD retinol level in the study sample was 1450 ± 720 ng/mL. The levels of retinol were deficient (<200 ng/mL) in one participant only (Table 1). No significant difference in retinol levels were found between males and females (*p* = 0.24), different BMI groups (*p* = 0.47), or between the participants with or without a history of chronic disease (*p* = 0.55) (Supplementary Figure S2).

### 3.2. Effect of the Demographic/Clinical Characteristics on the Response to the BNT162b2 Vaccine

The study sample was divided into two categories based on the previous exposure to SARS-CoV-2: exposed and unexposed. All the unexposed participants responded to the vaccine by producing detectable levels of the SARS-CoV-2 IgG antibodies 21 days after their first vaccine dose. There was a large variation in the response to the vaccine between the participants as the antibody levels ranged from 10 to 661 IU/mL on D21 with a mean level of 210 ± 150 IU/mL. There was no significant difference in the SARS-CoV-2 IgG antibody levels between males and females (*p* = 0.88), smokers and nonsmokers (*p* = 0.19), different BMI groups (*p* = 0.93), or between the participants with or without chronic diseases (*p* = 0.48) (Table 2).

The avidity of the D21 antibodies was also tested. The avidity index ranged from 4.8% to 72% with a mean ± SD avidity index of 34% ± 16%. Comparing the avidity indices between the different demographic/clinical groups showed no significant difference between males and females (*p* = 0.28), between BMI categories (*p* = 0.63), or between the participants with or without chronic diseases (*p* = 0.19) (Table 2).

In previously exposed participants, the mean antibody titer was 37 ± 23 IU/mL on D0. As expected, receiving the vaccine dose caused a significant increase in the antibody titer on D21 (mean ± SD = 223 ± 71, *p* value < 0.001). Compared to D0, the folds of the antibody level increase on D21 ranged from 1.1 to 32.6 folds (mean ± SD = 8.8 ± 7.3 folds). There was no significant difference in the titer increase between males and females, different BMI categories and participants with or without chronic diseases (Table 3).

### 3.3. Exploring the Effect of 25(OH)D and Retinol Levels on the Response to BNT162b2 Vaccine

**Figure 1:**
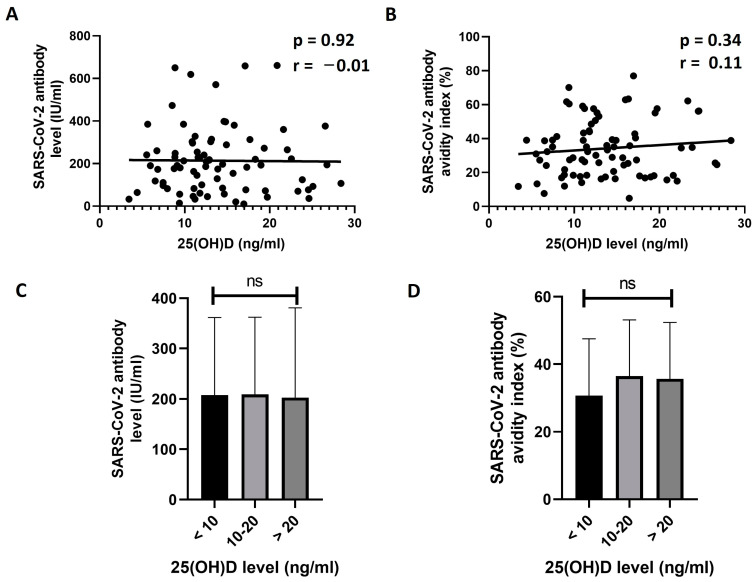
Analyzing the effect of vitamin D levels on SARS-CoV-2 antibody response in unexposed individuals. (**A**,**B**) show the correlation analysis between baseline (D0) 25(OH)D levels and the D21 level (**A**) or avidity index (**B**) of SARS-CoV-2 S1 IgG antibodies. No significant correlations were found (*p* > 0.05). In (**C**,**D**), the unexposed participants were divided into three groups based on the D0 levels of 25(OH)D, and the mean D21 antibody levels (**C**) or avidity indices (**D**) were compared between the groups. No significant differences in the levels or avidity were found between the groups (*p* > 0.05, One-way ANOVA). ns: not significant.

Next, the correlation between the 25(OH)D baseline level (D0) and the response to the vaccine in previously unexposed individuals was tested. No significant correlation was found between the D21 levels of SARS-CoV-2 IgG antibody and the D0 levels of 25(OH)D (r = −0.01, *p* = 0.92, Figure 1A). Similarly, the D0 25(OH)D level did not show a significant correlation with the avidity of the antibodies on D21 (r = 0.11, *p* = 0.34, Figure 1B). The previously unexposed participants were then divided into three status groups based on the level of 25(OH)D: less than 10 ng/mL, 10–20 ng/mL, and more than 20 ng/mL. The D21 antibody levels and avidity were then compared between the three groups. The results of this analysis showed no significant difference between the groups in the mean antibody level (*p* = 0.99, Figure 1C) or the avidity index (*p* = 0.40, Figure 1D).

**Figure 2:**
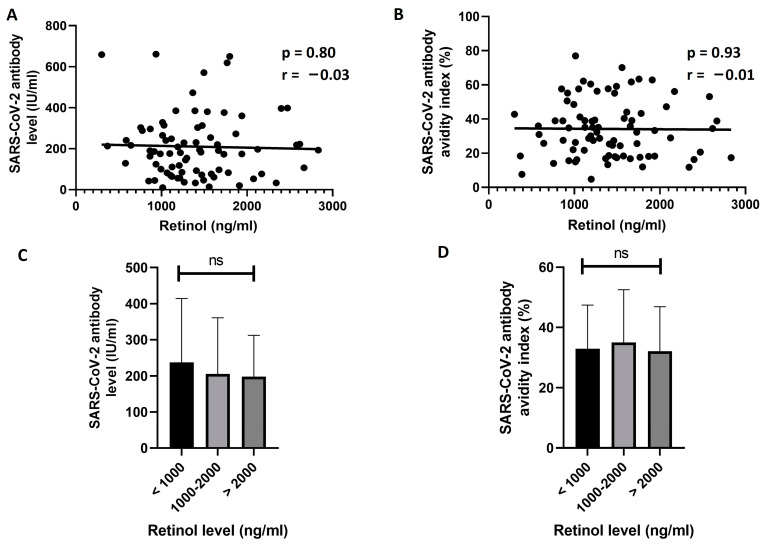
Analyzing the effect of vitamin A levels on SARS-CoV-2 antibody response in unexposed individuals. (**A**,**B**) show the correlation analysis between baseline (D0) retinol levels and the D21 level (**A**) or avidity index (**B**) of SARS-CoV-2 S1 IgG antibodies. No significant correlations were found (*p* > 0.05). In (**C**,**D**), the unexposed participants were divided into three groups based on the D0 levels of retinol, and the mean D21 antibody levels (**C**) or avidity indices (**D**) were compared between the groups. No significant differences in the levels or avidity of the antibodies were found between the groups (*p* > 0.05, One-way ANOVA). ns: not significant.

The effect of retinol level on responsiveness to vaccine in unexposed participants was then explored. The results of this analysis did not show any significant correlation between D0 retinol levels and D21 antibody titer (r = −0.03, *p* = 0.80, Figure 2A) or avidity (r = −0.01, *p* = 0.93, Figure 2B). Then, we divided the unexposed participants into three groups based on D0 levels of retinol: less than 1000 ng/mL, 1000–2000 ng/mL, and more than 2000 ng/mL. No significant difference between the groups was found in the mean D21 levels (Figure 2C) or avidity (Figure 2D) of SARS-CoV-2 S1 IgG antibodies (*p* = 0.72 and 0.81, respectively).

**Figure 3:**
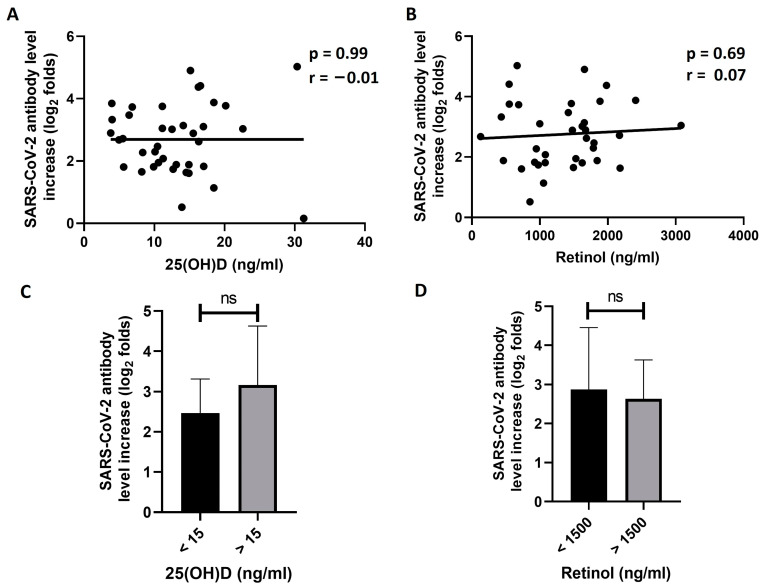
Analyzing the effects of vitamin D and vitamin A on SARS-CoV-2 antibody response in previously exposed individuals. Correlation between baseline (D0) levels of 25(OH)D (**A**) or retinol (**B**) and log_2_ folds of increase in SARS-CoV-2 S1 IgG antibody levels between D0 and D21 in previously exposed individuals. No significant correlation was found. In (**C**,**D**), exposed participants were dived into two groups of D0 25(OH)D levels (**C**) or two groups of D0 retinol levels (**D**). The means of log2 fold increase in SARS-CoV-2 S1 IgG antibody levels were compared between the groups. No significant difference in the antibody level increase was found between the groups (*p* > 0.05, *t* test). ns: not significant.

Then, we explored the effect of the two vitamins on the responsiveness to the vaccine in the participants who were previously exposed to the virus. To achieve this, log_2_ folds of the SARS-CoV-2 S1 antibody level increase between D0 and D21 were first calculated. Then, the correlation between the antibody increase and the baseline serum levels of 25(OH)D and retinol were investigated. The results of this analysis showed no significant correlation between the folds of antibody level increase and the baseline serum levels of 25(OH)D (r = −0.01, *p* = 0.99, Figure 3A). Similarly, baseline retinol serum levels did not significantly correlate with the antibody increase (r = 0.07, *p* = 0.69, Figure 3B). The previously exposed participants were then divided into two groups of baseline levels for each of the two vitamins. Dividing the participants into three groups, as in the previous analyses, was not feasible due to the relatively small size of the exposed participants. We selected a 25(OH)D level of 15 ng/mL and a retinol level of 1500 ng/mL as the cut-off values for dividing the participants into the groups. The means of the log_2_ folds of increase in SARS-CoV-2 S1 IgG antibody were then compared between the groups. The results showed a higher increase in the “more than 15 ng/mL 25(OH)D” group compared to the “less than 15 ng/mL 25(OH)D” group (Figure 3C). However, this difference was not statistically significant (*p* = 0.08). Similarly, there was no significant difference in the folds of antibody increase between the two groups of retinol levels (*p* = 0.59, Figure 3D).

**Figure 4:**
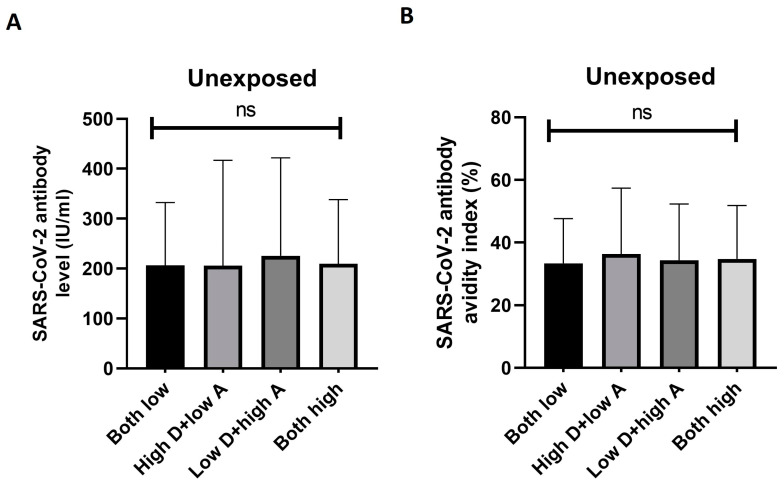
Analysis of the combined effect of vitamin D and vitamin A on the response to BNT162b2 vaccine. Unexposed participants were first divided into four groups based on the combined status of 25(OH)D and retinol. Then, the mean level (**A**) or avidity (**B**) of SARS-CoV-2 S1 IgG antibodies were compared between the four groups. No significant difference in the levels or avidity were found between the groups. ns: not significant.

Finally, we tested the effects of the two vitamins in conjunction with each other. To achieve this, the unexposed participants were distributed into four groups according to the combined status of the two vitamins. These groups were as follows: “Both low” group included participants with a 25(OH)D level of less than 15 ng/mL and a retinol level of less than 1500 ng/mL; “High D and low A” group contains participants with a 25(OH)D levels of more than 15 ng/mL and a retinol level of less than 1500 ng/mL; “Low D and high A” group contains participants with a 25(OH)D level of less than 15 ng/mL and a retinol level of more than 1500 ng/mL; and “Both high” group contains the participants with a 25(OH)D level of more than 15 ng/mL and a retinol level of more than 1500 ng/mL. Then, the mean levels and the avidity of SARS-CoV-2 S1 IgG antibodies were compared between the groups. The results of this analyses showed no significant differences in the mean antibody levels (*p* = 0.98, Figure 4A) or avidity (*p* = 0.95, Figure 4B) between the groups. A similar analysis was not conducted on the previously exposed participants due to its relatively small size, which does not allow for the separation of these participants into four representative vitamin status subgroups.

## 4. Discussion

The huge worldwide burden of the COVID-19 pandemic resulted in the development of COVID-19 vaccines in an unprecedented speed in human history. BNT162b2 (Pfizer/BioNTech) vaccine was the first COVID-19 vaccine to receive the approval of the health agencies in Jordan and many other countries. The efficacy of this vaccine was reported to be 95% in protection against COVID-19 in the initial clinical trial; however, variations in responsiveness between recipients were seen in real-world data [39,46]. Understanding the factors affecting the efficacy of this vaccine is considered as a priority to improve the protection against COVID-19 and for controlling SARS-CoV-2 spread. In this study, we explored the effect of vitamin D and vitamin A on antibody response to the first dose of the BNT162b2 vaccine. No significant correlation was found between the baseline levels of the two tested vitamins and the titer or avidity of generated SARS-CoV-2 antibodies three weeks after vaccination.

Previously, it was shown that the 1,25-dihydroxyvitamin D (1,25(OH)2D) form of vitamin D plays a role in activating innate immunity functions and modulating the inflammatory responses to multiple respiratory viral infections such as influenza, respiratory syncytial virus, and rhinovirus [47,48]. Furthermore, multiple observational reports have indicated that vitamin D level correlated with the susceptibility to a SARS-CoV-2 infection and the severity of COVID-19 [49,50,51]. Vitamin D deficiency influences the severity and outcomes of COVID-19 as part of the distinctive osteo-metabolic COVID-19 phenotype [52,53]. This phenotype is characterized by hypocalcemia, hypovitaminosis D with an inadequate compensatory response of the parathyroid hormones, and high rates of vertebral fractures. The usage of vitamin D supplements was reported to associate with a reduced risk of severe disease in SARS-CoV-2 infected individuals [54,55].

Likewise, vitamin A was also found to be crucial for maintaining normal immune functions. Vitamin A is important for the normal function of the mucosal immunity, development of B and helper T lymphocytes, and enhancing the antibody-mediated immunity [28,56]. Vitamin A supplementation was reported to improve clinical outcomes of pneumonia as vitamin A deficiency has been associated with severe pneumonia in Mycoplasma pneumoniae-infected children [57,58,59]. In addition, vitamin A could have a protective role against COVID-19 through several mechanisms including the inhibition of inflammation, modulating reactive oxygen species function, and shifting Th17 cells-mediated immunity toward regulatory T-cell phenotype [60,61]. Due to its effects on improving the disease outcomes of children’s pneumonia, vitamin A was proposed as a candidate therapeutic agent against SARS-CoV-2 infections [62].

It is not clear whether vitamin D and vitamin A play a role in promoting immune response to COVID-19 vaccines. Previously, several in vivo studies reported that antibody production and mucosal immunity improve in mice models after the co-administration of 1,25-(OH)2D3 with the polio vaccine, the *Haemophilus influenzae* type b conjugate vaccine, and the subunit hepatitis B surface antigen vaccine [63,64,65,66]. Thus, the usage of vitamin D as an adjuvant to improve vaccination effectiveness was suggested for some vaccines [67]. Similarly, vitamin A supplementation was reported to improve the responsiveness to rabies and pneumococcal vaccines [32,68]. Therefore, this study was conducted to investigate the effect of the two key vitamins, vitamin D and vitamin A, on the responsiveness to the BNT162b2 COVID-19 vaccine.

The mean serum levels of 25(OH)D in the study population was 14.3 ± 7.4 ng/mL. This level falls in the vitamin D deficiency category, as the widely accepted cut-off value for vitamin D deficiency is 20 ng/mL [42]. In fact, the levels of vitamin D were deficient in 85% of the study population according to this classification. This finding is consistent with several previous studies that reported extremely high prevalence of vitamin D deficiency among the Jordanian population. For example, El-Khateeb et al. reported that approximately 90% of the Jordanian population have a low vitamin D status [69]. In a second study from Jordan, Abu-Samak et al. found that 91% of the study sample had 25(OH)D levels of less than 30 ng/mL [70]. Our results showed significantly higher levels of 25(OH)D in males compared to females. A similar result was reported previously in Jordan by Al-Horani et al. [71]. This result could be attributed to differences in the frequency of outdoor activities and dress style between Jordanian males and females.

Our results demonstrated variations in antibody responsiveness to the BNT162b2 vaccine between participants. This variation was not associated with gender, smoking status, BMI, or the existing chronic diseases. There was no significant correlation between the baseline vitamin D levels and the SARS-CoV-2 antibody response in previously exposed or unexposed participants. This result, in addition to the results of most of the previous studies, provide evidence that baseline levels of vitamin D have no effect on the short-term response to the BNT162b2 vaccine [17,18,19,20]. However, Zelini et al. and di Filippo et al. reported a long-term effect of the baseline levels of vitamin D on the persistence of the response [19,20]. Another two studies found no significant associations between vitamin D levels at time points after the second dose of the vaccine and the response to the vaccine [72,73]. The effects of baseline levels of vitamin D were not investigated in these two studies. Contrary to our results, one group reported a positive effect of vitamin D on the peak response to one dose of the vaccine, which was achieved at 3.2 weeks after vaccination on average [21]. Although the cause of this discrepancy is not clear, it could be attributed to differences in the timing of the compared antibody levels (i.e., peak response vs. a specific time-point in our study) and to diffrences in the charecteristics of the studied samples.

Our results showed that a single vaccination of previously exposed individuals induced a significant increase in the antibody titer on D21 compared to D0 (mean increase = 8.8 ± 7.3 folds, *p* < 0.0001). A similar response to COVID-19 vaccines in previously infected individuals was reported by other studies [74,75,76,77]. Moreover, boosting the infection-acquired immunity with the COVID-19 vaccine was shown to generate a more persistent immunity compared to the vaccination alone [78]. Many groups reported that previously infected vaccine recipients generate higher antibody responses than the uninfected vaccine recipients [74,75,76,77]. Our data showed slightly higher D21 antibody levels in the exposed group compared to the unexposed group; however, the difference between the two groups was not significant. The cause of this unexpected result is not clear to us, and it could be attributed to the relatively small size of the exposed group, the race of the studied population, the variant of the virus that caused the previous infections, the timing of the infections as well as other possible factors.

In this study, baseline retinol levels had no significant effect on the responsiveness to the BNT162b2 vaccine in both previously exposed and unexposed recipients’ groups. To the best of our knowledge, this study is the first to investigate the effect of vitamin A on the responsiveness to COVID-19 vaccines. The effect of vitamin A on the outcomes of COVID-19 was investigated in a pilot randomized clinical trial [79]. This clinical trial found no effect of vitamin A supplementation on the severity outcomes of COVID-19 in hospitalized patients.

The results of current study could help in understanding the effects of vitamin D and vitamin A on the antibody response to the BNT162b2 vaccine. However, this study has some limitations that should be addressed. First, the sample size of this study was 124 participants. This sample provided good insights into the effects of the two vitamins; however, a larger sample would have more representative results. Second, the majority of the study participants showed low levels of vitamin D and normal levels of vitamin A. For this reason, the results of this study reflect the effects of the two vitamins within these ranges. It is worth it to mention here that similar high ratios of vitamin D deficiency in the Jordanian population were reported by many studies before, making the study sample a good representation of vitamin D status among Jordanians [69,70]. Nevertheless, future studies with a more diverse sample in the levels of the two vitamins will give a better understanding of the effect of the two vitamins on the response to the vaccine. Future studies are also needed to investigate the effects of other vitamins and micronutrients on the responses to COVID-19 vaccines, including vitamin C, vitamin E, and zinc. Moreover, future studies are needed to understand the effects of vitamins and micronutrients on other vaccine platforms such as the inactivated virus vaccines, subunit vaccines, and viral vector vaccines.

## Figures and Tables

**Table 1:** Characteristics of the study sample.

Characteristic	Value
Number	124
Age (mean ± SD)	43.8 ± 9.2
Sex	
Female	59 (48%)
Male	65 (52%)
BMI category	
Normal	42 (34%)
Overweight	43 (35%)
Obese	39 (34%)
Chronic diseases	
One or more chronic disease	26 (21%)
Hypertension	20 (16%)
Diabetes	14 (11%)
Cardiac diseases	6 (4.8%)
Exposure to SARS-CoV-2 virus prior to vaccination	
Exposed	39 (32%)
Unexposed	85 (69%)
25(OH)D level (ng/mL) (mean ± SD)	14.3 ± 7.4
Deficient	108 (85%)
Insufficient	15 (12%)
Normal	4 (3.1%)
Retinol (ng/mL) (mean ± SD)	1450 ± 720
Deficient	1 (0.8%)
Normal	123 (99.2%)

**Table 2:** Effect of participants’ demographic/clinical characteristics on the responsiveness to BNT162b2 vaccine in previously unexposed individuals.

Characteristic	Number	SARS-CoV-2 Ab Titer (Mean ± SD)	*p* Value	Avidity Index (%) (Mean ± SD)	*p* Value
Sex			0.88		0.28
Male	47	210 ± 170		32 ± 17	
Female	38	210 ± 140		36 ± 15	
BMI category			0.93		0.63
Normal	30	220 ± 190		36 ± 18	
Overweight	31	200 ± 120		34 ± 15	
Obese	24	210 ± 140		32 ± 17	
Chronic diseases			0.48		0.19
No	65	220 ± 160		34 ± 16	
Yes	20	190 ± 130		35 ± 19	

**Table 3:** The effect of demographic/clinical factors on the responsiveness to BNT162b2 vaccine in previously exposed individuals.

Characteristic	Number	SARS-CoV-2 Ab Titer Increase (log_2_ Folds)	*p* Value
Gender			0.63
Male	17	2.8 ± 1.1	
Female	21	2.6 ± 1.1	
BMI category			0.54
Normal	12	3.0 ± 1.2	
Overweight	11	2.7 ± 1.1	
Obese	15	2.5 ± 1.1	
Chronic diseases			0.93
No	31	2.7 ± 1.2	
Yes	6	2.7 ± 0.6	

## Data Availability

All the data used to support the results reported in this study are available from the corresponding author upon reasonable request.

## Supplementary Materials

The following supporting information can be downloaded at https://www.mdpi.com/article/10.3390/vaccines11091509/s1: Figure S1: Comparing 25(OH)D levels between sex, BMI, and chronic diseases groups; Figure S2: Comparing retinol levels between sex, BMI, and chronic diseases groups.
