# Supplementary material for: Exploring the Effects of Vitamin D and Vitamin A Levels on the Response to COVID-19 Vaccine
Source: Vaccines (Basel). 2023 Sep 21;11(9):1509. doi: 10.3390/vaccines11091509 (PMC10535137; doi:10.3390/vaccines11091509)
Supplement: Supplementary file 1 [file vaccines-11-01509-s001.zip › vaccines-2579630-supplementary.pdf]

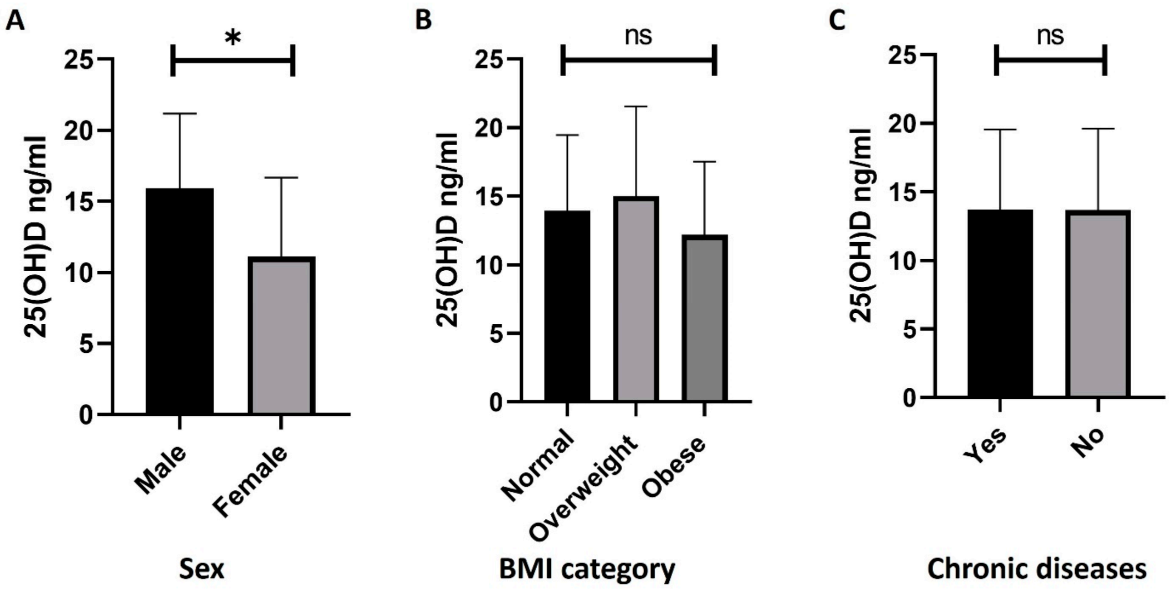

Figure S1: **Analyzing the effect of sex (A), BMI (B), and chronic diseases (C) on the levels of 25(OH)D.** In **A**, the mean level of 25(OH)D level was significantly higher in males compared to females ($p < 0.001$, t test). In **B** and **C**, no significant differences in the mean 25(OH)D levels were found between the BMI groups or the chronic diseases history groups. (*) $p < 0.05$, (ns) not significant.

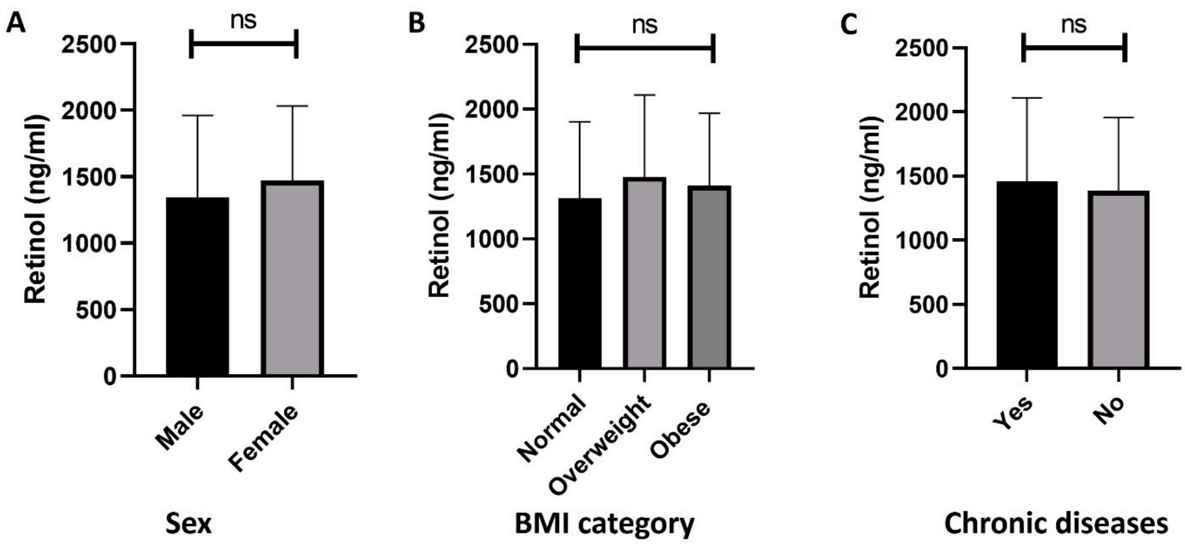

Figure S2: **Analyzing the effect of sex (A), BMI (B), and chronic diseases (C) on the levels of retinol.** No significant differences in the mean retinol levels were found between males and females, BMI groups or the chronic diseases history groups ($p > 0.05$). ns: not significant
